# Non-alcoholic fatty liver disease and steatohepatitis: State of the art on effective therapeutics based on the gold standard method for diagnosis

**DOI:** 10.1016/j.molmet.2020.101049

**Published:** 2020-07-13

**Authors:** Maryam Mahjoubin-Tehran, Antonio De Vincentis, Dimitri P. Mikhailidis, Stephen L. Atkin, Christos S. Mantzoros, Tannaz Jamialahmadi, Amirhossein Sahebkar

**Affiliations:** 1 Student Research Committee, Mashhad University of Medical Sciences, Mashhad, Iran; 2 Department of Medical Biotechnology, Faculty of Medicine, Mashhad University of Medical Sciences, Mashhad, Iran; 3 Clinical Medicine and Hepatology Unit, Campus Bio-Medico University of Rome, via Alvaro del Portillo, 200, 00128 Rome, Italy; 4 Department of Clinical Biochemistry, Royal Free Hospital Campus, University College London Medical School, University College London (UCL), London, United Kingdom; 5 Weill Cornell Medicine Qatar, Doha, Qatar; 6 Department of Medicine, Beth Israel Deaconess Medical Center, Harvard Medical School, Boston, MA, USA; 7 Section of Endocrinology, Boston VA Healthcare System, Harvard Medical School, Boston, MA, USA; 8 Biotechnology Research Center, Pharmaceutical Technology Institute, Mashhad University of Medical Sciences, Mashhad, Iran; 9 Department of Food Science and Technology, Quchan Branch, Islamic Azad University, Quchan, Iran; 10 Department of Nutrition, Faculty of Medicine, Mashhad University of Medical Sciences, Mashhad, Iran; 11 Halal Research Center of IRI, FDA, Tehran, Iran; 12 Neurogenic Inflammation Research Center, Mashhad University of Medical Sciences, Mashhad, Iran; 13 Biotechnology Research Center, Pharmaceutical Technology Institute, Mashhad University of Medical Sciences, Mashhad 9177948564, Iran; 14 Polish Mother’s Memorial Hospital Research Institute (PMMHRI), Lodz, Poland

**Keywords:** Non-alcoholic fatty liver disease, Liver biopsy, Non-alcoholic steatohepatitis

## Abstract

**Objective:**

The prevalence of non-alcoholic fatty liver disease and non-alcoholic steatohepatitis (NAFLD/NASH) is increasing. NAFLD/NASH may progress to cirrhosis and hepatocellular carcinoma. However, most patients with NAFLD/NASH will die from a vascular cause. There are no approved pharmacological treatments for NASH/NAFLD. Many clinical trials have been, or are being, undertaken; however, the challenge is the assessment of the clinical endpoint. The main objective of this narrative review was to evaluate the efficacy of drugs used in clinical trials for the treatment of NAFLD/NASH that included a liver biopsy as the gold standard.

**Methods:**

A literature search was conducted using 3 databases (PubMed, Scopus, and Google Scholar) to identify the clinical trials that included liver biopsy assessment before and after treatment.

**Results:**

Interventional clinical trials (n = 33) involving 18 different agents, alone and in combination, were identified. Pioglitazone is the only agent that has shown consistent benefit and efficacy in clinical trials. Pentoxifylline, rosiglitazone, and ursodeoxycholic acid had both positive and negative results from clinical trials. There is also evidence for vitamin E and metformin. Other drugs, including bicyclol, cysteamine bitartrate, l-carnitine, liraglutide, obeticholic acid, oligofructose, selonsertib, silymarin, and statins, each had a single clinical study.

**Conclusions:**

In summary, the available molecules demonstrated a significant improvement in NASH and/or liver fibrosis in a minority of patients; thus, other drugs should be identified, possibly those acting on alternative pathophysiological pathways, and tested for their safety and efficacy.

## Highlights

• There are no currently approved pharmacological treatments for NASH/NAFLD.
• Confirmation of effective therapies for NAFLD/NASH is challenging due to the limitations of non-biopsy methods.
• We reviewed the efficacy of drugs used in NAFLD/NASH trials that included a liver biopsy as the gold standard.

## 1 Introduction

Non-alcoholic fatty liver disease (NAFLD) is an increasing global public health problem and a common cause of chronic liver disease [1] (the worldwide prevalence of NAFLD is estimated at approximately 25%) [2]. The prevalence of NAFLD is increasing in parallel with the increase in type 2 diabetes mellitus (T2DM) and obesity, and NAFLD is predicted to affect >30% of the adult population of the United States (US) [3].

The classical definition of NAFLD is hepatic steatosis in the absence of other liver disease [4]. In NAFLD, fat accumulates in the liver as a result of increased free fatty acid delivery to the liver, increasing triglyceride synthesis, decreasing triglyceride export, and reducing beta-oxidation [5]. Patients with NAFLD commonly have insulin resistance (IR) that enhances lipolysis from adipose tissue [5]. Liver biopsy in NAFLD shows hepatic steatosis without inflammation or hepatocellular injury (hepatocyte ballooning) [6]; however, between 10% and 25% of patients with NAFLD show inflammatory infiltration leading to non-alcoholic steatohepatitis (NASH). Approximately 25% of patients with simple steatosis, may progress to NASH in 3 years [4]. NASH is characterized by hepatic steatosis and lobular inflammation accompanied by hepatocyte injury (e.g., in the form of ballooning) in the presence or absence of fibrosis [6]. The exact prevalence of NASH is currently unknown because a liver biopsy is necessary for a definitive diagnosis [4]. NAFLD/NASH can progress to cirrhosis, HCC (hepatocellular carcinoma), and can an indication for liver transplantation [7]. NASH increases the risk of liver-related morbidity and mortality, as well as chronic kidney disease, cardiovascular disease, and cancer [2,3]. Of concern is that the progression from NAFLD to NASH is more aggressive and rapid in children than in adults [2].

Despite the increasing number of patients, there are limited therapeutic approaches and no approved drug treatments for NAFLD and NASH [7]. In recent years, an increasing number of emerging therapies have undergone clinical evaluation [8]. In this context, an important challenge in the field of assessing NASH/NAFLD therapeutics is to accurately assess the response to treatment [7]. The gold standard to determine the progression or regression is a liver biopsy [9]. Noninvasive tests such as hepatic enzymes, imaging, NAFLD fibrosis score, Fibroscan, and FibroMeter may help NASH diagnosis by identifying fibrosis [2,5,10] but remain surrogate markers.

## 2 Search strategy

For this narrative review, randomized controlled trials for the treatment of NAFLD and/or NASH that have used liver biopsy assessment before and after treatment were identified by using 3 databases: PubMed, Scopus, and Google Scholar. The key words were (“non-alcoholic fatty liver disease” OR “non-alcoholic fatty liver disease” OR “non-alcoholic steatohepatitis” OR “non-alcoholic fatty liver disease” OR NAFLD OR NASH OR “fatty liver”) AND (biopsy OR histology OR histopathology OR histopathologic OR histological OR histopathologic OR histopathological) AND (trial OR “clinical trial” OR “randomized controlled trial;” Table 1). We excluded studies based on a liver biopsy performed only at the beginning of the trial and not as an endpoint evaluation. We only included agents with evidence of efficacy based on histological outcomes (Table 2). Clinical trials on agents showing an absence of histological efficacy were excluded. Only articles written in the English language were included.

**Table 1:** Characteristics of liver biopsy-based randomized controlled trials in non-alcoholic liver disease and non-alcoholic steatohepatitis.

Agent	Population (Type & Number)	Comparator group	Age	Dose	Treatment duration (Week)	Effect^a^	Adverse events^d^	Ref.
Bicyclol	NAFLD with IFG 31	Vitamin E 100 mg 3 times daily	Adults	25 mg 3 times daily	24	+	Mild abdominal distension and dizziness	[12]
Cysteamine Bitartrate (CB)	NAFLD activity scores of 4 or higher 169	Placebo	Adolescents	twice daily (300–450 mg) age dependent	52	+^b^	Gastrointestinal adverse events	[16]
Docosahexaenoic Acid Plus Vitamin D	NAFLD and vitamin D deficiency 43	Placebo	Children and adolescents	daily DHA (500 mg) plus vitamin D (800 IU) once daily	24	+^c^	–	[19]
Docosahexaenoic acid-choline-vitamin E	NASH 40	Placebo	Children and adolescents	combining 250 mg of DHA, 39 UI of vitamin E and 201 mg of choline	24	+^c^	–	[20]
l-Carnitine	NASH 74	Placebo	Adults	2 g/day	24	+	Nausea, moderate headache, and abdominal pain	[23]
Liraglutide	NASH 52	Placebo	Adults	subcutaneous injections of liraglutide (1∙8 mg daily)	48	+	Diarrhea, constipation, and loss of appetite	[29]
Metformin	NAFLD 55	Vitamin E OR prescriptive weight-reducing diet	Adults	2 g/day	48	+^c^	–	[32]
Metformin	NAFLD 48	Placebo	Adults	500 mg/day increased every week until 2500 mg or 3000 mg	24	–	–	[30]
Metformin	NASH 19	Placebo diet and exercise	Adults	500 mg daily	48	–	–	[33]
Metformin	NAFLD 173	Placebo	Adolescents	Daily dosing 1000 mg of metformin	96	+	Nausea, vomiting, and diarrhea	[34]
Obeticholic acid	NASH 283	Placebo	Adults	25 mg daily	72	+	Pruritus	[37]
Obeticholic acid	NASH 931	Placebo	Adults	10 or 25 mg daily	72	+	Pruritus	[38]
Pentoxifylline	NASH 30	Placebo	Adults	400 mg 3 times daily	48	–	Headache and abdominal cramps	[43]
Pentoxifylline	NASH 55	Placebo	Adults	400 mg 3 times a day	48	+	Nausea	[44]
Pioglitazone	type 2 diabetes and NASH 55	Placebo	Adults	45 mg daily	24	+	Fatigue and mild lower-extremity edema	[46]
Pioglitazone	NASH 74	Placebo	Adults	30 mg/day	48	+	Fluid retention	[47]
Pioglitazone	NASH without diabetes 247	Placebo	Adults	30 mg daily	96	+	–	[48]
Pioglitazone	NASH 101	Placebo	Adults	45 mg/d	72	+	Weight gain∗	[49]
Prebiotic (Oligofructose)	NASH 14	Placebo	Children and adolescents	8 g/day for 12 weeks followed by 16 g/day for 24 weeks	36	+	–	[51]
Prebiotic (*Bifidobacterium longum* with fructo-oligosaccharides)	NASH 66	Placebo and lifestyle modification	Adult	*Bifidobacterium longum* with fructo-oligosaccharides 2.5 g	24	+	–	[52]
Rosiglitazone	NASH 33	Placebo	Adults	4 mg/day for the first month and 8 mg/day thereafter	48	+	Weight gain∗	[54]
Rosiglitazone	NASH 53	Placebo	Adults	8 mg/day	96	–	Asthenia, muscular cramps, swollen legs and weight gain	[55]
Rosiglitazone	NASH 137	4 mg of rosiglitazone and 500 mg of metformin twice daily or 4 mg of rosiglitazone twice daily and 50 mg of losartan once daily	Adults	4 mg twice daily	48	–	–	[56]
Selonsertib	NASH and stage 2 or 3 liver fibrosis 72	125 mg of simtuzumab with or without selonsertib	Adults	6 or 18 mg of selonsertib once daily	24	+	Headache, nausea, sinusitis, nasopharyngitis, upper abdominal pain, back pain, and fatigue.	[57]
Silymarin	NASH and a NAFLD activity score 4 or more 99	Placebo	Adults	700 mg, 3 times daily	48	+	Ureteric calculi	[58]
Silymarin (Legalon®)	NASH without cirrhosis with NAS ≥4	Placebo	Adults	420 mg, 700 mg, 3 times daily	48	–	Ureteric calculi	[59]
Ursodeoxycholic Acid	NASH 166	Placebo	Adults	between 13 and 15 mg/kg/d	96	–	Gastroin-testinal adverse events	[60]
Ursodeoxycholic acid	NASH 185	Placebo	Adults	23–28 mg/kg/day	72	+	Diarrhea∗	[61]
Vitamin E with Ursodeoxycholic Acid	NASH 48	UDCA with vitamin E OR UDCA with placebo OR placebo/placebo	Adults	12–15 mg · per kg per day with vitamin E 400 IU twice a day	96	+	–	[62]
Vitamin E with pioglitazone	NASH 20	vitamin E (400 IU/day)	Adults	vitamin E (400 IU/day) and pioglitazone (30 mg/day)	24	+	–	[63]
Vitamin E	NASH without diabetes 247	Placebo	Adults	800 IU daily	96	+	Weight gain∗	[48]
vitamin E	NAFLD 173	Placebo	Adolescents	daily dose of 800 IU of vitamin E	96	+	–	[34]
Cenicriviroc	NASH, with NAS≥ 4, and liver fibrosis stages of 1–3 252	Placebo	Adults	150 mg daily	48	+^b^	Arrhythmia	[64]
Elafibranor	NASH without cirrhosis	Placebo	Adults	80–120 mg daily	52	+^b^	mild increase in serum creatinine levels∗	[65]
Statin	NASH 107	untreated	Adults	–	24	+	–	[74]
Ezetimibe	NAFLD 32	untreated	Adults	10 mg/day	24	+	–	[80]
Resmetirom	NASH 125	Placebo	Adults	80 mg/day	36	+	Transient mild diarrhea and nausea∗	[39]

**DHA**: Docosahexaenoic Acid, **IFG**: impaired fasting glucose**, NAFLD**: non-alcoholic fatty liver disease, **NAS**: NAFLD activity score, **NASH**: non-alcoholic steatohepatitis**, UDCA**: Ursodeoxycholic acid.

a Positive effect of drugs defined as an improvement in at least in 1 histological feature.

b Although there was no difference between groups in the primary outcome, patients receiving CBDR had significant improvement in secondary outcomes.

c Biopsy at the end of therapy was performed only in the treatment group for ethical reasons.

d Adverse events did not differ by treatment group except those marked with a star (∗).

**Table 2:** Changes in histological features of the liver with different therapeutic agents.

Agent	Steatosis	Fibrosis	Hepatocyte ballooning	Lobular inflammation	NAS	Ref
Bicyclol	–	–	–	↓	↓	[12]
Cysteamine Bitartrate (CB)^a^	–	–	–	↓	–	[16]
l-carnitine	↓	↓	N	↓	N	[23]
Liraglutide a	↓	–	↓	–	–	[29]
Metformin	–	–	↓	–	–	[34]
Vitamin E	–	–	↓	–	↓	[34]
Obeticholic acid	↓	↓	↓	↓	↓	[37]
Obeticholic acid	–	↓	↓^a^	↓^a^	–	[38]
Pentoxifylline	↓	↓	–	↓	↓	[44]
Pioglitazone	↓	↓	↓	↓	↓	[46] [48] [49]
Prebiotic (Oligofructose)	↓	–	–	–	↓	[51]
Prebiotic (*Bifidobacterium longum* with fructo-oligosaccharides)	↓	–	N	–	↓	[52]
Rosiglitazone^a^	↓	–	–	–	–	[54]
Selonsertib^a^	↓	↓	–	↓	↓	[57]
Silymarin	–	↓	–	–	–	[58]
UDCA	–	–	↓	–	–	[61]
DHA Plus Vitamin D b	↓	–	↓	↓	↓	[19]
DHA Plus Vitamin E & choline b	↓	–	↓	–	↓	[20]
UDCA Plus Vitamin E	↓	–	–	–	–	[62]
Pioglitazone Plus Vitamin E	–	–	↓	↓	–	[63]
Cenicriviroc	–	↓^a^	–	–	–	[64]
Statin	↓	↓	–	–	–	[74]
Ezetimibe	–	↓	↓	–	–	[80]
Resmetirom	–	–	–	–	↓	[39]

**DHA**: Docosahexaenoic Acid, **NAS**: NAFLD activity score**, UDCA**: Ursodeoxycholic acid.

N: Not determined.

↑: Increase.

↓: Decrease.

–: Not significantly effected.

a Data derived from percentage of “patients with improvement” in histological parameters.

b Results of treatment at the end of the study compared with the baseline because the liver biopsy at the end of the study was performed in the active group alone for ethical reasons.

In the included studies, the NAFLD activity score (NAS) was defined as the unweighted sum of the scores for steatosis (0–3), lobular inflammation (0–3), and ballooning (0–2); this score ranged from 0 to 8. A decrease in HOMA-IR (log homeostasis assessment model analysis for IR) score represented improved insulin sensitivity. The NASH activity index represented the sum of scores for parenchymal inflammation (0–4), cellular injury (0–4), and steatosis. Liver tests reported included alanine transaminase (ALT), aspartate transaminase (AST), alkaline phosphatase (ALP), gamma glutamyl transpeptidase (GGT), albumin, and bilirubin (Table 3).

**Table 3:** Alterations in liver function indices with different therapeutic agents.

	ALT	AST	ALP	GGT	Albumin	Bilirubin	Ref
Bicyclol	↓	–	N	–	N	N	[12]
Cysteamine Bitartrate (CB)	↓	↓	–	↓	N	N	[16]
l-carnitine	↓	↓	N	↓	–	N	[23]
Liraglutide	–	–	–	↓	–	–	[29]
Metformin	↓	↓					[32]
Obeticholic acid	↓	↓	↑	↓	–	↓	[37,38]
Pentoxifylline	↓	–	N	N	N	N	[44]
Pioglitazone	↓	↓	↓	↓	↓	–	[46,47] [48,49]
Prebiotic (*Bifidobacterium longum* with fructo-oligosaccharides)	–	↓	N	N	–	–	[52]
Selonsertib	↓	↓	N	↓	N	N	[57]
UDCA	↓	–	N	↓	N	N	[61,62]
DHA Plus Vitamin D	↓	↓	N	–	N	N	[19]
DHA Plus Vitamin E & choline	↓	–	N	–	N	N	[20]
UDCA Plus Vitamin E	↓	↓	N	N	N	N	[62]
Elafibranor	↓	N	↓	↓	N	N	[65]
Resmetirom	↓	↓	–	↓	–	–	[39]

N: Not determined.

↑: Increase.

↓: Decrease.

–: Not significantly affected.

**ALP**: Alkaline phosphatase, **ALT**: Alanine transaminase, **AST**: Aspartate transaminase, **DHA**: Docosahexaenoic Acid**, GGT**: Gamma glutamyl transpeptidase, **UDCA**: Ursodeoxycholic acid.

## 3 Bicyclol

Bicyclol is a derivative of dimethyl-4, 4′-dimethoxy-5, 6, 5′, 6′-dimethylene dioxybiphenyl-2, 2′-dicarboxylate (DDB), a synthesized analog of traditional Chinese medicine from the herb *Fructus Schizandrae*. Bicyclol may be effective for treating chronic hepatitis B and C viral infections (in China) and protect against lipid injury and oxidation [11].

Patients (n = 31) with NAFLD and impaired fasting glucose (IFG) were enrolled in a randomized open label controlled trial of bicyclol *versus* vitamin E. After lifestyle changes and a daily dose of 1500 mg/day of metformin, the treatment groups received either bicyclol (25 mg 3 times daily) or vitamin E (a-tocopherol; 100 mg 3 times daily for 24 weeks). Steatosis, inflammation, hepatocellular ballooning, and NASs decreased in both groups after treatment. However, decreases in histopathological inflammation (−1.25 vs. 0.6) and NAS (−2.68 vs. 1.94) in the bicyclol group were significantly improved compared with vitamin E. In addition, bicyclol significantly reduced serum ALT activity (62.6 vs. 51.87 U/L) compared with the vitamin E group. In this study, 1.79% of the patients who received bicyclol reported abdominal distension and mild diarrhea during the study, and 1.8% of patients in the control group reported mild abdominal distension and dizziness. There were no abnormal laboratory results related to either study drugs [12].

## 4 Cysteamine bitartrate (CB)

Cysteamine (β-mercapto-ethylamine) bitartrate (CB) is an approved drug in the United States and European Union for nephropathic cystinosis in adults and children [13]. Cysteamine is a sulphydryl compound that can prevent paracetamol-induced hepatic necrosis and liver damage in paracetamol poisoning [14,15].

In a randomized placebo-controlled double-blinded trial, for 52 weeks, 169 children with NAFLD activity scores ≥4 received either cysteamine bitartrate delayed release (CBDR) or placebo twice daily (300 mg for those weighing 65 kg, 375 mg for those weighing >65–80 kg, and 450 mg for those weighing >80 kg). The primary outcome was a decrease in the NAS of ≥2 points without worsening fibrosis; the secondary outcome was any decrease in histological features. There were significantly more patients showing an improvement in lobular inflammation in the CBDR group than in the placebo (36 vs. 21%). In a *post hoc* analysis of children weighing ≤65 kg, those taking CBDR had a 4-fold better chance of histological improvement. Although there was no significant difference between groups in the primary outcome measure, patients receiving CBDR had significant changes in the secondary outcomes, with a reduction in the mean activities of serum ALT (−53 vs. −8 U/L), AST (−31 vs. −4 U/L), and GGT (−10 vs. −1) compared with placebo. Other biochemical parameters did not differ between groups [16].

## 5 Docosahexaenoic Acid (DHA)

DHA acid may be effective in liver steatohepatitis because it can decrease liver triglycerides in NAFLD [17,18].

In a randomized, double-blind placebo-controlled trial, 43 children with NAFLD who were obese and had a vitamin D deficiency received 500 mg DHA plus 800 IU vitamin D daily or placebo for 12 months. The major limitation of this study was that only the treatment group had a liver biopsy at the end of the study due to ethical reasons. DHA plus vitamin D treatment reduced the NAS (from 5.40 to 1.92), steatosis (from 2.25 to 1.0), ballooning (from 1.6 to 0.46), lobular inflammation (from 1.5 to 0.88), and portal inflammation (from 1.6 to 1.0). In addition, DHA and vitamin D improved AST (−8.55 vs. 0 U/L) and ALT (−15.75 vs. 7.75 U/L) compared with the placebo group. Moreover, triglycerides, low-density lipoprotein cholesterol (LDL-C), and body mass index (BMI) decreased in the treatment group together with a persistent, significant increase in vitamin D levels. None of the treated patients developed hypercalcemia and/or nephrotoxicity, and no adverse events were reported [19].

In another randomized placebo-controlled clinical trial involving children with NASH, 40 participants received lifestyle modification plus placebo, or lifestyle modification plus a mix containing 250 mg of DHA, 39 UI of vitamin E, and 201 mg of choline every day for 6 months. All patients were recommended to follow a hypocaloric diet (25–30 kcal/kg/day) and engage in a twice-weekly 1-h physical activity during the treatment, and for a further 6 months of follow-up. The limitation of this trial was that the end of the study, liver biopsy was only performed in the active treatment group for ethical reasons. Significant improvements in steatosis (1.05 vs. 1.85), ballooning (1.35 vs. 0.60) and NAS (4.35 vs. 2.65) were found at the end-of-study liver biopsy compared with baseline. Severe steatosis (grade 3) was significantly decreased from 50% to 5% of patients. Significant improvements in ALT (from 53.5 to 35.3 IU/L) and fasting glucose levels were observed only in the treatment group. No adverse events were reported [20].

## 6 l-carnitine

l-carnitine is a quaternary amine that may prevent the development of NASH [21]. l-carnitine has been demonstrated to limit oxidative stress, reduce lipid levels, and control inflammatory responses [22]; furthermore, it mediates the transport of long-chain fatty acids across the mitochondrial membrane. Thereby, l-carnitine facilitates the removal of fatty acids accumulating in mitochondria that lead to the unbalanced hepatic fat turnover resulting in steatosis [23,24].

In a randomized, controlled clinical trial, 74 patients with NASH received 2 l-carnitine 1 g tablets plus diet or placebo plus diet per day at the same dosage and regimen for 24 weeks. l-carnitine caused a reduction in steatosis (−2.28 vs. −1.11), hepatocellular injury (−1.95 vs. −1.19), portal inflammation (−1.49 vs. −1.07), fibrosis (−1.31 vs. −0.85), and NASH activity index (6.23 vs. −3.63) compared with placebo. Each of the component features of the NASH activity index (steatosis, parenchymal inflammation, and hepatocellular injury) improved significantly. The mean NASH activity score decreased from 9.42 to 3.19. Overall, 86% of patients had improvement in fibrosis scores, and 97% of patients had a histological response. The biochemical parameters AST (−71.7 vs. −46.1 IU/L), ALT (−58.4 vs. −37.4 IU/L), and GGT (−37.6 vs. 20.4 IU/L) were also significantly improved compared with placebo. In addition, compared with placebo, the patients in the l-carnitine group showed significant improvements in total cholesterol, LDL-C, plasma glucose, HOMA-IR, C-reactive protein (CRP), and tumor necrosis factor (TNF)-α [23].

## 7 Liraglutide

Liraglutide, an FDA (US Food and Drug Administration)-approved medication for treating T2DM, is a long-acting analog of human glucagon-like peptide-1 (GLP-1) [25,26]. GLP-1 is an incretin hormone that induces insulin secretion and decreases glucagon secretion [26]. In addition, GLP-1 decreases energy intake and body weight by prolonging gastric emptying and inducing satiety [26]. There is an association between NAFLD and metabolic syndrome that increases the risk of T2DM, dyslipidemia, and obesity [27]. Furthermore, liraglutide was shown to have anti-inflammation activity [28]. Therefore, GLP-1 receptor analog therapy may have potential for the treatment of NAFLD and patients with NASH. GLP-1 receptors are present in hepatocytes, and it was shown that liraglutide may directly reduce liver fibrosis and steatosis in an *in vivo* study [26].

In a multicenter, double-blinded, randomized, placebo-controlled phase 2 trial of subcutaneous injections of liraglutide, 52 patients who were overweight with histological evidence of NASH received 1.8 mg daily liraglutide or placebo for 48 weeks. There were significantly more patients showing an improvement in hepatocyte ballooning (61 vs. 32%, p = 0·05) and steatosis (83 vs. 45%, p = 0·009) in the liraglutide group compared with the placebo group. Indeed, 39% of patients in the liraglutide group versus 9% of patients in the placebo group (p = 0·019) had a resolution of histologically defined NASH. Fewer patients in the liraglutide group showed progression of fibrosis compared with placebo (9 vs. 36%, p = 0·04). Serum GGT activity was significantly reduced in the liraglutide group compared with the placebo group (−33.7 and −7.2 U/L, p = 0.010). Most adverse events were grade 1 (mild) to grade 2 (moderate) in severity and were transient. These events were similar in the 2 treatment groups, except for gastrointestinal disorders that were more frequent with liraglutide including nausea, diarrhea, and abdominal pain [29].

## 8 Metformin

Metformin, an insulin sensitizer used to treat DM, may be a promising option for NAFLD [30]. The action of metformin as an antidiabetic agent is through decreasing gluconeogenesis in the liver, increasing the uptake of glucose in the muscle, enhancing oxidation of fatty acids in adipose tissue, and improving insulin sensitivity [31].

In an open label, randomized trial, 55 patients with NAFLD who were nondiabetic received 2 g/day metformin for 12 months. The control group received 800 IU vitamin E (n = 28) or a weight-reducing diet. The important limitation of this study was that at the end of the study, liver biopsy was conducted only in the metformin group for ethical reasons. Histological assessment showed a significant decrease (compared with baseline) in the necroinflammation score (from 1.88 to 1.23, p = 0.012), fibrosis score (from 2.88 to 2.18, p = 0.012), and NASH index (from 6.53 to 4.47, p<0.0001). Treatment with metformin significantly improved serum ALT and AST activities (compared with vitamin E and placebo). The number of cases with a normal ALT at the end of the study in the metformin group was greater than in the diet group and in the vitamin E group. In the metformin group (*vs*. the diet group) fasting glucose, insulin, and HOMA were significantly reduced. No side effects were reported [32].

In a controlled trial, 48 patients with NAFLD were randomly assigned to either metformin or placebo for 6 months. Individuals received 1 tablet (500 mg metformin or placebo) per day, followed by weekly titrations until a maximal daily dose of 2500 mg or 3000 mg (if body weight was ≥90 kg) was reached after 4 or 5 weeks. No differences between the metformin and placebo groups were observed for liver steatosis, NAS-score, liver transaminases or markers of insulin resistance, or inflammation. Changes in serum activities of ALT and AST did not differ between the groups. By contrast, beneficial effects of metformin were observed in changes in body weight, serum levels of cholesterol, LDL-C, glucose, and HbA_1c_. Two patients in the metformin group dropped out of the study because of gastrointestinal complications and incidence of exanthema [30].

A prospective randomized placebo-controlled trial evaluated the effects of diet, exercise, and placebo compared with diet, exercise, and metformin for 12 months in 19 nondiabetic patients with IR and NASH. Both groups received dietary recommendations for weight loss and exercise 4 times/week. The treatment group received long-acting metformin (500 mg/day; titrated to 1000 mg/day). There were no differences between the 2 groups for steatosis, ballooning, intra-acinar/portal tract inflammation, fibrosis, and NAS. There were, however, significant improvements in steatosis and NAS across all study subjects. ALT activities decreased by 40.7 IU/L in the placebo group, 21.5 IU/L in the treatment group, and 31.6 IU/L overall AST activities decreased by 20.1 IU/L, 5.7 IU/L, and 13.2 IU/L, respectively. However, the differences between the 2 groups did not differ for ALT, AST, ALP, or other biochemical parameters [33].

In a randomized, double-blind, double-dummy, placebo-controlled trial conducted in 173 NAFLD children and adolescents, participants received 800 IU of vitamin E or 1000 mg of metformin or placebo daily for 96 weeks. The ballooning degeneration score was significantly improved in the metformin group (−0.3 vs. 0.1) and vitamin E group (−0.5 vs. 0.1) compared with placebo. NAS (−1.8 vs. 0.7) was significantly reduced and resolution of NASH (58 vs. 28%) was significantly increased in the vitamin E group compared with placebo. Serum biochemistry parameters did not differ between the metformin group and vitamin E groups compared with placebo. For those taking metformin, adverse effects included dose-dependent nausea, vomiting, and diarrhea, although the reported severity or frequency of adverse events between treatment groups was not significant. Five children in the placebo group, 1 in the metformin group, and none in the vitamin E group developed diabetes, but this difference was not statistically significant [34].

## 9 Obeticholic acid (OCA)

Obeticholic acid (OCA; 6α-ethyl-chenodeoxycholic acid) is a bile acid analog of CDCA (chenodeoxycholic acid) with a 100-fold higher affinity, compared with CDCA, for the farnesoid X receptor (FXR) [35]. FXR is a promising target for NAFLD therapy because it is a nuclear receptor that plays several roles, including regulation of lipid metabolism and modulation of liver growth [35]. OCA has anti-cholestatic and hepato-protective properties [36].

In a phase 2, multicenter, double-blind, placebo-controlled, parallel group, randomized clinical trial, 283 non-cirrhotic NASH participants received 25 mg daily OCA orally or placebo for 72 weeks. Histological assessment showed significant improvement (treatment vs. placebo group) in fibrosis (35 vs. 19%, p = 0.03), hepatocellular ballooning (46 vs. 31%, p = 0.030), steatosis (61 vs. 38%, p = 0.001), and lobular inflammation (53 vs. 35%, p = 0.006). The scores for fibrosis (−0·2 vs. 0·1, p = 0.010), hepatocellular ballooning (−0·5 vs. −0·2, p = 0·030), steatosis (−0·8 vs. −0·4, p = 0·0004), lobular inflammation (−0·5 vs. −0·2, p = 0·0006), and NAS (−1.7 vs. −0.7, p < 0·0001) were significantly decreased by OCA compared with placebo. Furthermore, compared with placebo, treatment with OCA significantly improved ALT (−38 vs. −18 U/L, p < 0·0001), AST (−27 vs. −10 U/L, p = 0·0001), GGT (−37 vs. −6 U/L, p < 0·0001) activities, and bilirubin (−1 vs. 0.6 μmol/L, p = 0·002). However, OCA treatment increased total cholesterol and LDL-C and decreased high-density lipoprotein cholesterol (HDL-C) compared with placebo. Clinical adverse events were generally mild to moderate in severity and were similar in the 2 groups for all symptoms except pruritus. Pruritus was reported in 23% of OCA-treated patients and 6% of placebo-treated patients [37].

In a phase 3 randomized placebo-controlled trial, 931 patients with NASH and severe fibrosis received OCA 10 mg/day, OCA 25 mg/day, or placebo for 18 months. The primary endpoints were either fibrosis improvement with no worsening of NASH or NASH resolution with no worsening of liver fibrosis. Results showed that once-daily OCA 25 mg achieved 1 primary endpoint (fibrosis improvement with no worsening of NASH) in 23% of participants (p = 0·0002) and the other primary endpoint was not fulfilled. Patients in this group showed improvements in hepatocellular ballooning (35% compared with placebo, p = 0·001) and lobular inflammation (44% compared with placebo, p = 0·032). Pruritus, was the most common adverse event that affected 51% of the patients in OCA 25 mg/day group, 28% of the OCA 10 mg/day treatment group, and 19% of the placebo group [38].

## 10 Resmetirom

Resmetirom is a liver-targeted agent that binds thyroid hormone receptor-β to counteract the toxicities associated with thyroid hormone excess (largely mediated through thyroid hormone receptor-α) [39]. Resmetirom could improve NASH *via* enhancing hepatic fat metabolism and attenuating lipotoxicity [39].

In a randomized, double-blind, placebo-controlled study, NASH patients in fibrosis stage 1–3, (N = 125) received resmetirom (MGL-3196) or placebo 80 mg/day for 36 weeks. Results showed that resmetirom reduced hepatic fat compared with placebo (−37·3 vs. −8.5%, p < 0·0001). NAS was significantly reduced in the treatment group compared with the placebo group. The proportion of patients with a ≥2-point reduction in NAS with at least a 1-point reduction in ballooning or inflammation was significantly greater in the treatment group compared with placebo (46% vs. 19%, p = 0·017). Furthermore, resmetirom significantly reduced ALT, AST, and GGT compared with placebo [39].

## 11 Pentoxifylline (PTX)

PTX, a methylxanthine derivative, is a non-selective phosphodiesterase inhibitor that causes vasodilatory effects [40]. PTX was reported to decrease inflammation by inhibiting the production of TNFα that is recognized to promote inflammatory reactions in the development of NAFLD [41]. PTX was initially used in the treatment of intermittent claudication and then for the treatment of peripheral artery disease and liver injuries such as alcoholic hepatitis and NASH [42].

In a randomized controlled trial, 30 patients with NASH received 1,200 mg PTX or placebo for 12 months. Both histological and biochemical features did not differ between groups. Adverse events were mild and most frequently headache and abdominal cramps and did not differ between groups [43].

In another randomized placebo-controlled trial, 55 biopsy-confirmed patients with NASH received 400 mg PTX 3 times/day or placebo for 1 year. Treatment significantly improved steatosis score (−0.85 vs. −0.04, p < 0.001), lobular inflammation (−0.45 vs. 0.08, p = 0.023), fibrosis (−0.2 vs. 0.4, p = 0.038), and NAS (−1.6 vs. −0.1, p < 0.001) compared with placebo. An improvement of 30% or more in ALT activity from baseline was observed in the treatment (57%) compared with the placebo group (23%), p = 0.016. Adverse effects were similar in both groups, and the common adverse events were nausea and vomiting [44].

## 12 Pioglitazone

Pioglitazone is used as an antidiabetic agent [45]. Pioglitazone is a thiazolidinedione that targets insulin resistance and adipose tissue dysfunction that cause liver lipotoxicity in fatty liver disease [3]. Pioglitazone acts by binding to the PPARγ (peroxisome proliferator-activated receptor gamma) that plays a key role in lipid metabolism and glucose regulation [45].

In a placebo-controlled trial, 55 patients with impaired glucose tolerance or T2DM and NASH received a hypocaloric diet (a reduction of 500 kcal/day) plus 45 mg pioglitazone daily or a hypocaloric diet plus placebo for 6 months. Histological improvement in the pioglitazone group was significantly more than that in the placebo group: steatosis (65 vs. 38%, p = 0.003), ballooning (54 vs. 24%, p = 0.02), lobular inflammation (65 vs. 29%, p = 0.008), and necroinflammation (85 vs. 38%, p = 0.001). Treatment (compared with placebo) significantly decreased AST activity (−9 vs. −19 U/L, p = 0.04) and ALT activity (−39 vs. −21 U/L, p < 0.001). Furthermore, pioglitazone lowered triglycerides, fasting plasma glucose, and insulin levels. Mild edema and fatigue developed in 1 subject who received pioglitazone, and no other adverse effect were observed [46].

In another randomized, placebo-controlled trial, 74 nondiabetic patients received a standard diet, exercise, and either 30 mg/day pioglitazone or placebo for 12 months. Histological features including hepatocellular injury (p = 0.005), Mallory-Denk bodies (p = 0.004), and fibrosis (p = 0.05) were reduced in the pioglitazone group compared with placebo. ALT (−37.7 vs. −6.9 U/L, p = 0.009) and GGT (−121.7 vs. −6 U/L, p = 0.002) activities were reduced in the treatment group compared with the placebo group. No adverse events were observed in the pioglitazone treatment group compared with the placebo group [47].

In a randomized placebo-controlled trial, 247 patients with NASH without DM received 30 mg pioglitazone daily, 800 IU vitamin E, or placebo for 96 weeks. Compared with placebo, treatment with pioglitazone and vitamin E significantly improved steatosis (69 and 54 vs. 31%, p < 0.001, p = 0.005), lobular inflammation (60 and 54 vs. 35%, p = 0.004, p = 0.02), hepatocellular ballooning (NS and 50 vs. 29%, p = 0.08, p = 0.01), and NAS (−1.9 and −1.9 vs. −0.5, p < 0.001, p < 0.001), with a resolution of NASH (47 and 36 vs. 21%, p = 0.001, p = 0.05) in pioglitazone and vitamin E compared with placebo, p value for pioglitazone versus placebo, and p value for vitamin E versus placebo, respectively. Furthermore, serum biochemical features significantly improved in the pioglitazone and vitamin E versus the placebo group, respectively: ALT (−40.8 and −37.0 vs. −20.1 U/l, p < 0.001, p = 0.001), AST (−20.4 and −21.3 vs. −3.8 U/l, p < 0.001, p < 0.001), GGT (−21.1 and −14.0 vs. −4.0 U/l, p < 0.001, p = 0.003), and ALP (−12.0 and −9.3 vs. −3.8, p = 0.004, p = 0.008) activity (p value for pioglitazone vs. placebo and p value for vitamin E vs. placebo, respectively) [48].

In another randomized, double-blind, placebo-controlled trial, 101 patients with prediabetes or T2DM and NASH received either 45 mg/d pioglitazone or placebo for 18 months. Treatment improved the steatosis score (−1.1 vs. −0.2, p < 0.001), inflammation (−0.6 vs. −0.1, p < 0.001), ballooning (−0.6 vs. −0.2, p = 0.001), and fibrosis (−0.5 vs. 0, p = 0.039) compared with placebo. Resolution of NASH in the pioglitazone group (51%) was significantly greater than with placebo (19%), p < 0.001. The percentage of patients that had a ≥2-point reduction in NAS without worsening of fibrosis was significantly greater in the pioglitazone group than in the placebo group, p < 0.001. Compared with placebo, pioglitazone treatment significantly decreased AST (p = 0.001) and ALT p < 0.001 activities and decreased triglyceride (p = 0.018), HDL-C levels (p < 0.001), and liver fat content (p < 0.001). The common adverse events were musculoskeletal, respiratory/otolaryngologic, and gastrointestinal, and there was no difference between the pioglitazone and placebo groups [49].

## 13 Prebiotics

Gut microbiota dysbiosis is considered a contributing factor to NASH development. Prebiotics are substrates selectively used by host microorganisms. Prebiotics alter the gut microbiota by increasing the growth and activity of health-promoting bacteria [50]. Oligofructose is a prebiotic that enhances *Bifdobacterium* and reduces *Clostridium* clusters XI and I [51]. In addition, oligofructose can lower serum triglycerides, cholesterol, and very low-density lipoproteins [50].

A randomized trial evaluated the effects of *Bifidobacterium longum* with fructo-oligosaccharides in the treatment of NASH. Patients (n = 66) received *B. longum* with fructo-oligosaccharides (2.5 g) and lifestyle modification (i.e. diet and exercise) or placebo and lifestyle modification for 24 weeks. There was a significant reduction in steatosis (2.22 vs. 1.5, p < 0.05) and the NAS (6.22 vs. 4.29, p < 0.05) compared with placebo. Moreover, treatment significantly reduced AST (−69.6 vs. −45.9 IU/mL - the authors used IU/mL within the abstract and text and IU/L within the table, but the correct units are probably IU/dL - p < 0.05), LDL-C (−0.84 vs. −0.18 mmol/L, p < 0.001), CRP (−2.9 vs. −0.7 mg/L, p < 0.05), TNF-α (−0.45 vs. −0.12 ng/mL, p < 0.001), HOMA-IR (−1.1 vs. −0.6, p < 0.001), and serum endotoxin (−45.2 vs. −30.6 pg/mL, p < 0.001) [52].

In a placebo-controlled, randomized pilot trial 14 patients with NASH (NAS ≥5) received oligofructose (8 g/day for 12 weeks followed by 16 g/day for 24 weeks) or placebo. Prebiotic therapy significantly decreased steatosis and NAS compared with placebo. Treatment did not alter ALT, ALP, and GGT activities. There were no adverse events from consuming oligofructose [51].

## 14 Rosiglitazone

Rosiglitazone, an antidiabetic drug, improves insulin sensitivity [53]. IR leads to fat accumulation in the liver and the development and progression of steatohepatitis. As such, rosiglitazone may be useful in the treatment of NASH by reversing IR [54]. However, evidence of its increasing the risk of cardiovascular events has caused its withdrawal in many countries and limited its use.

In a placebo-controlled trial, 63 patients with biopsy-proven NASH were randomly assigned to either rosiglitazone (4 mg/day for the first month followed by 8 mg/day thereafter) or placebo for 1 year. More patients treated with rosiglitazone than receiving placebo had significantly improved steatosis (47 vs. 16%) and normalized transaminase levels (38 vs. 7%). There were no improvements in other histological parameters. The main adverse effect was weight gain (mean gain of 1.5 kg in the rosiglitazone group vs. 1 kg in the placebo group; p < 0.01), and the main reason for dose reduction/discontinuation was the incidence of painful, swollen legs [54].

In a randomized trial, 53 patients with NASH received 8 mg/day rosiglitazone or placebo for 2 years. There was no difference in the biochemical parameters or histological features [55].

In another randomized controlled trial, 137 patients with NASH received rosiglitazone 4 mg twice daily, rosiglitazone 4 mg and 500 mg metformin twice daily, or rosiglitazone 4 mg twice daily and losartan 50 mg once daily for 48 weeks. Serum aminotransferases were reduced in all 3 groups but did not differ between groups. There was no difference between treatment groups for all of the histological parameters. No difference between treatment groups for adverse events was detected [56].

## 15 Selonsertib

Activation of apoptosis signal-regulating kinase 1 (ASK1) in the setting of oxidative stress can lead to an activation of stress response pathways that worsens hepatic apoptosis, inflammation, and fibrosis. Therefore, selonsertib, a selective inhibitor of ASK1, could be useful for the treatment of NASH [57].

In a multicenter randomized control trial, 72 patients with NASH received either 6 or 18 mg of selonsertib orally once daily with or without once-weekly injections of 125 mg of simtuzumab or simtuzumab alone for 24 weeks. Simtuzumab is a humanized monoclonal antibody against the lysyl oxidase-like molecule 2, an enzyme involved in the extracellular matrix remodeling through the crosslinkage of collagen and elastin. Due to the absence of an effect of simtuzumab on histological parameters, selonsertib groups with and without simtuzumab were pooled. Treatment significantly improved histological parameters, with a reduction in fibrosis (43%, 30%, and 20%), patients with progression to cirrhosis (3%, 7%, and 20%), patients with ≥1 point reduction in NAS (52%, 41%, and 60%), patients with ≥2 point reduction in NAS (23%, 19%, and 20%), steatosis ≥1 point reduction (32%, 30%, and 20%), lobular inflammation ≥1 point reduction (32%, 22%, and 20%), and ballooning ≥1 point reduction (16%, 33, and 30%) in the selonsertib 18 mg ± simtuzumab group, selonsertib 6 mg ± simtuzumab group, and simtuzumab group, respectively. Compared with the baseline, treatment reduced serum ALT (−8, −6, and −3 U/L), AST (−5, −4, and −3 U/L), and GGT (−7, −2, and −2 U/L) activities in the selonsertib 18 mg ± simtuzumab group, selonsertib 6 mg ± simtuzumab group, and simtuzumab group, respectively. Moreover, the triglyceride (−21, 12, and −30 mg/dL), total cholesterol (−10, −5, and −13 mg/dL), HDL-C (−2, 1, and 2 mg/dL), LDL-C (−10, −5 and −25 mg/dL), and HOMA-IR (0.98, 2.17, and −0.22) levels were significantly changed in the selonsertib 18 mg ± simtuzumab group, selonsertib 6 mg ± simtuzumab group, and simtuzumab group, respectively. The highest number of adverse events in the selonsertib groups were headache and nausea [57].

## 16 Silymarin

Silymarin is a mixture of flavonolignans and polyphenolic compounds derived from the milk thistle plant, *Silybum marianum*, used for the treatment of liver disease. Silymarin has anti-inflammatory, antifibrotic, and antioxidant properties that may be beneficial in patients with NAFLD [58].

In a randomized, double-blind, placebo-controlled trial, 99 biopsy-proven patients with NASH and NAS ≥4 received 700 mg silymarin or placebo 3 times/day for 48 weeks. Compared with placebo, treatment with silymarin significantly improved fibrosis in patients (fibrosis change: –0.184 in silymarin group vs. +0.100 placebo group, p = 0.026). Triglyceride levels were significantly improved in the silymarin group (−0.20 vs. +0.04 mmol/L, p = 0.017). There were no significant differences in adverse events and discontinuations in the silymarin and placebo groups [58].

In a multicenter double-blind placebo-controlled trial, the effect of standardized silymarin preparation (Legalon®) was tested. Legalon® is a proprietary milk thistle seed extract standardized to a silymarin content of 140 mg/capsule. Patients with NASH, without cirrhosis, and with NAS ≥4 (n = 78) received 420 mg or 700 mg of Legalon® or placebo 3 times per day for 48 weeks. The histological improvement between groups was not significantly different. However, improved steatosis and lobular inflammation in the Legalon® group was more than that for the palcebo group but a statistically significant histological improvement was no observed. There were no significant differences in adverse events among the treatment groups [59].

## 17 Ursodeoxycholic acid (UDCA)

UDCA is a natural bile acid with several hepatoprotective activities [60]. UDCA reduces oxidative stress and has antiapoptotic effects that may benefit patients with NAFLD/NASH [61].

In a randomized clinical trial, 166 patients with NASH received between 13 and 15 mg/kg/day of UDCA or placebo for 2 years. There was no difference between the UDCA and placebo groups in biochemical or histological features. A trend toward a higher incidence of gastrointestinal adverse events in the UDCA compared with the placebo group was observed; however, the rate of clinical adverse events was similar in both groups [60].

In a double-blind, randomized, placebo-controlled trial, 185 patients with NASH received 23–28 mg/kg/day UDCA or placebo for 18 months. Treatment with UDCA significantly improved lobular inflammation (−0.51 vs. −0.19 in placebo). However, other histopathological features did not differ between groups. Compared with placebo, GGT activity significantly improved in the UDCA group (−52.42 vs. −16.84 U/L). Diarrhea was the side effect in the UDCA group (11 in UDCA group vs. 1 in placebo group). No patient dropped out because of adverse effects of UDCA [61].

In a randomized placebo-controlled trial, 48 patients with NASH received UDCA (12–15 mg/kg/day) plus vitamin E (400 IU twice a day; UDCA/Vit E), UDCA with placebo (UDCA/P), or placebo/placebo (P/P) for 2 years. Steatosis was improved in the UDCA/Vit E group (p < 0.05). None of the histological parameters were altered in the UDCA/P group. There were significant decreases in the ALT and AST activities in the UDCA/Vit E group (p < 0.05) and the ALT activity in UDCA/P group (p < 0.05). Vitamin E and UDCA appeared safe, and their combination was well tolerated with no patient dropouts as a result of side effects [62].

## 18 Vitamin E

Oxidative stress is implicated in NASH pathogenesis. Therefore, vitamin E as an antioxidant may be effective for the treatment of NASH [63].

Twenty nondiabetic and non-cirrhotic subjects with NASH received vitamin E alone (400 IU/day) versus vitamin E (400 IU/day) and pioglitazone (30 mg/day). Combination therapy produced a significant decrease in steatosis cytological ballooning, Mallory's hyaline, and inflammation, compared with vitamin E alone. Both groups were similar with respect to AST, ALT, and ALP activities. Combination therapy of pioglitazone and vitamin D significantly increased the metabolic clearance of glucose and decreased circulating fasting free fatty acid (FFA) and insulin levels [63].

## 19 Cenicriviroc (CVC)

CVC is a dual antagonist of chemokine receptor (CCR) types 2 and 5. Its anti-antifibrotic and inflammatory effects are mediated by CCR2 and CCR5 blockade. CVC has demonstrated antifibrotic activity in animal models of liver and renal fibrosis [64].

A randomized, double-blind, placebo-controlled trial assessed CVC for the treatment of NASH with liver fibrosis. Patients with NASH, NAS ≥4, and liver fibrosis stages 1–3 received CVC 150 mg or placebo orally for 1 year. The primary outcome was defined as a NAS improvement (2 points) with no worsening fibrosis. Secondary outcomes were defined as a resolution of steatohepatitis with no worsening of fibrosis; fibrosis improvement by 1 stage with no worsening of steatohepatitis was observed. The primary endpoint did not differ between the CVC and placebo groups; however, the fibrosis improvement and no worsening of steatohepatitis (% subjects who achieved improvement in fibrosis) were significantly greater in the CVC group compared with placebo (20 vs. 10%, p = 0.023). Tolerability and safety of CVC were comparable with placebo [64].

## 20 Elafibranor

Elafibranor is a peroxisome proliferator-activated receptor-α (PPARα) and peroxisome proliferator-activated receptor-δ (PPARδ) dual agonist. PPARδ agonists have shown efficacy in improving liver histology in NASH. Elafibranor improves lipid metabolism and insulin sensitivity and reduces inflammation [65].

Ratziu et al. evaluated the safety and efficacy of elafibranor in a randomized, double-blind placebo-controlled trial; 276 patients with NASH without cirrhosis received elafibranor 80 mg, elafibranor 120 mg, or placebo daily for 52 weeks. The primary outcome was no fibrosis worsening; however, this did not differ between the elafibranor and placebo groups. A greater proportion of subjects with a resolution of NASH without worsening fibrosis was observed in the 120 mg elafibranor group compared with the placebo group (19% vs. 12%). Liver enzymes, lipids, and markers of systemic inflammation were reduced in the elafibranor 120 mg group. Elafibranor was well tolerated but produced a mild increase in serum creatinine levels [65].

## 21 Statins

In addition to the well-known cholesterol-lowering effect, statins are reputed for the lipid-independent pleiotropic effects that justify their use in different patient populations not necessarily having hypercholesterolemia [[66], [67], [68], [69], [70], [71], [72]]. Statins may be recommended in patients with NAFLD/NASH for their lipid-lowering, antioxidant, and anti-inflammatory effects, as well as a decrease in the associated increased cardiovascular risk [73]. Well-conducted clinical trials to verify their effect on liver inflammation and fibrosis have not been conducted. However, a large observational cross-sectional multicenter study showed that statins were in subjects with NAFLD, with no hepatotoxic effect, and with beneficial effects on steatosis, NASH, and fibrosis [74]. A small prospective study with no control arm of 20 subjects with NASH, metabolic syndrome, and dyslipidemia treated with rosuvastatin monotherapy for 12 months also showed a benefit [75]. This effect may be partially explained by a reduction in tumor necrosis factor-α (TNF-α) levels; TNF-α is known to play a role in the pathogenesis of NASH [68]. In conclusion, in line with current guidelines [76], statins may be prescribed in NAFLD subjects to treat dyslipidemia, prevent cardiovascular risk, and have beneficial effects on the liver [77,78]. No specific indications are available on which statin or dose should be prescribed.

## 22 Ezetimibe

Ezetimibe is an LDL-C lowering agent, which can be considered a safe option for lipid lowering in patients with NAFLD [79]. In a randomized controlled trial, the effect of ezetimibe (10 mg/day) in combination with a standard energy diet and exercise was tested in 32 patients with NAFLD for 6 months. Fibrosis stage and ballooning score were improved with ezetimibe treatment. However, ezetimibe increased hepatic long-chain fatty acids and HbA_1c_; Thus, further evaluation is necessary [80].

## 23 Conclusions

**Figure 1:**
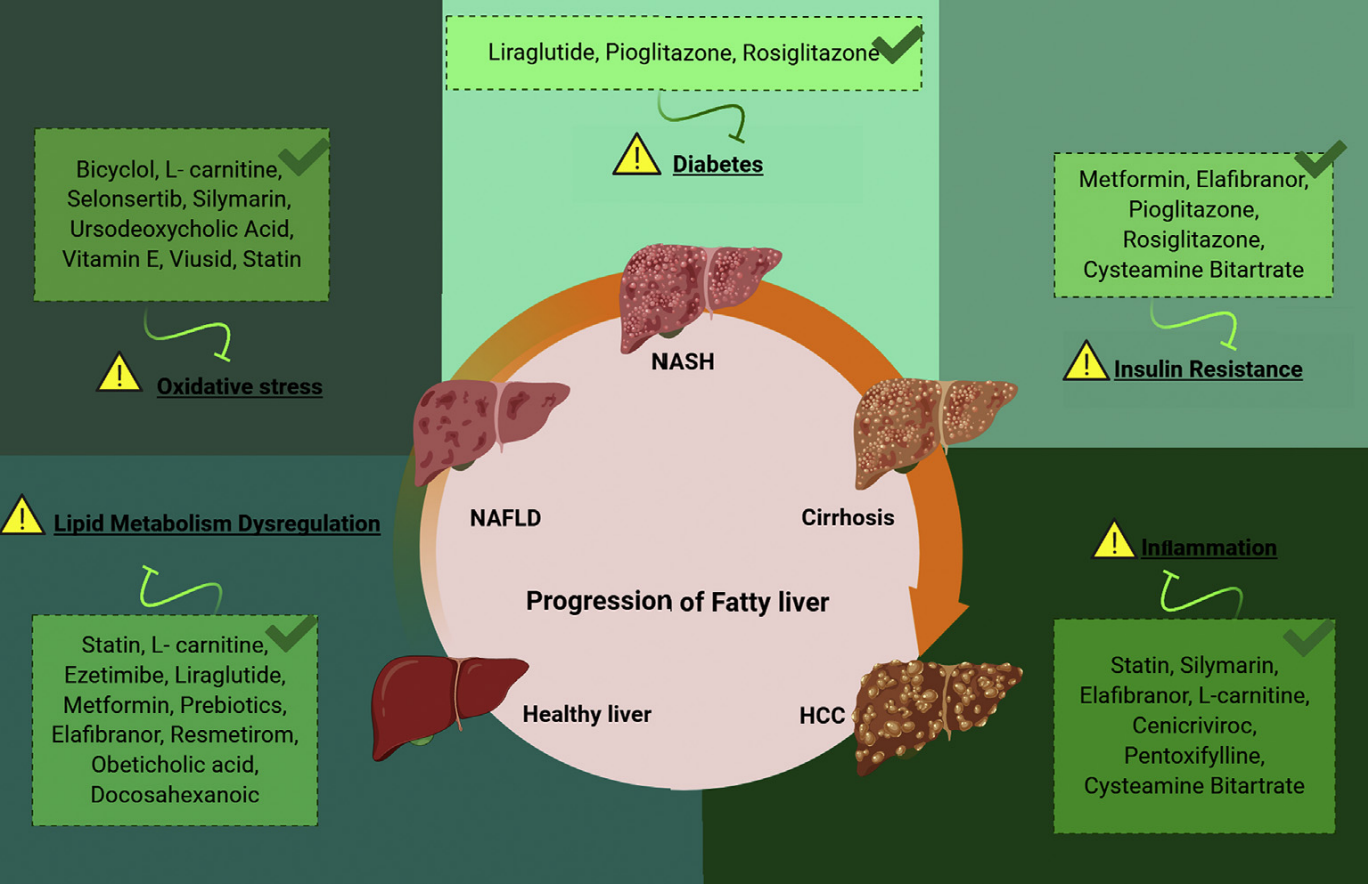
Possible mode of action of agents used in clinical trials for non-alcoholic fatty liver disease and steatohepatitis that used pre-treatment and post-treatment liver biopsy as the endpoints.

NAFLD has become a growing public health problem with no licensed therapeutic agents. The cornerstone of current management is dietary and lifestyle intervention to achieve weight loss, along with the optimization of metabolic risk factors, such as diabetes mellitus and dyslipidemia. However, these goals are difficult to implement mainly because of poor adherence. Therefore, in selected cases, the off-label use of medications with demonstrated effects on NASH histological features can be considered. Insulin sensitizers, such as pioglitazone and liraglutide, and hepatoprotective agents, such as vitamin E, may be the preferred options in clinical practice. The optimal duration of these therapeutic trials has not been established, and no firm recommendations are available; thus, the current management of the more severe patients (i.e., those with NASH and advanced liver fibrosis) is mainly left to the individual experience of treating physicians and local practice [6,76,81]. To bridge this gap, many clinical trials have been conducted with different therapeutic agents and promising results in some cases. The different pathophysiological pathways involved in NAFLD/NASH improvement are presented in Figure 1. Of these drugs, those with evidence of efficacy based on liver biopsy are of particular importance (Table 1). Pioglitazone and vitamin E have shown benefits for NASH histological features and are the only recommended agents in current clinical guidelines [6,76]. Pentoxifylline and ursodeoxycholic acid have both positive and negative results from clinical trials and require further clarification. Similar results have been observed for rosiglitazone, whose prescription is hampered by its withdrawal in many countries. Despite its effect in improving IR, 2 meta-analysis concluded against any effect of metformin on liver histology of patients with NAFLD and NASH [82,83].

Bicyclol, cysteamine bitartrate, l-carnitine, liraglutide, obeticholic acid, oligofructose, selonsertib, silymarin, and statins were part of one clinical study each; thus, further confirmation of their efficacy is necessary. The same level of evidence also applies to viusid, a nutritional supplement comprising glycyrrhizic acid, ascorbic acid, and zinc that has been shown to improve histological indices of NAFLD in a single randomized, controlled trial with a follow-up of 6 months [84].

The stages of clinical development of the aforementioned drugs are as follows: rosiglitazone (NCT00492700, NCT00492700), ursodeoxycholic acid (NCT00470171), cysteamine bitartrate (NCT00799578), elafibranor (NCT01694849), cenicriviroc (NCT02217475), and l-carnitine (NCT01617772) are in phase 2; pentoxifylline (NCT00267670), vitamin E (NCT00655018), metformin (NCT00303537), obeticholic acid (NCT02548351, NCT03439254), and selonsertib (NCT03053063, NCT03053050) are in phase 3; and pioglitazone and silymarin (NCT02973295) are in phase 4 (NCT00994682, NCT00227110). Although some drugs such as metformin and pioglitazone have received approval (for other diseases), other agents must be evaluated for their safety in addition to the efficacy for NAFLD/NASH. Therefore, new approved therapeutic agents for the treatment of NAFLD/NASH might be available soon.

However, after appraising the available literature, some critical points deserve consideration. First, the majority of the presented drugs are supported by a single clinical trial. As such, further investigations are necessary to confirm their effect on NAFLD/NASH, and to date, many of them cannot be considered with optimism regarding their early introduction into clinical practice. Second, even when multiple trials have been conducted, the sample of enrolled subjects is generally limited and short treatment periods have often been tested. Greater (>300–400 subjects) and more prolonged (>36–48 months) trials should be designed; they should include repeated liver biopsies during treatment, to retain adequate statistical power to determine histological outcomes. These trials should also accurately record long-term adverse effects. In this regard, excessive concerns regarding the ethical impracticability of liver biopsies for assessing inclusion and efficacy criteria should be considered in relation to the projected burden of NAFLD/NASH.

Finally, based available data, the most promising drug seems to be OCA, which showed a significant improvement in liver fibrosis in 18% (10 mg dose group) and 23% (25 mg dose group) of subjects in the interim analysis of its phase 3 trial. However, the relevant proportion of subjects experiencing moderate to severe pruritus (28% and 51% for the 10 mg and 25 mg dose groups, respectively) leaves concerns regarding its real practice tolerability.

In summary, an observation is that even in the best scenarios, the available molecules demonstrated a significant improvement in NASH and/or liver fibrosis in a minority of patients; thus, other drugs should be identified, possibly those acting on alternative pathophysiological pathways, and tested for their safety and efficacy. Additionally, the field of long noncoding RNAs should be examined because of the novel insights into their role in NASH and liver fibrosis development [85].

The focus of this review was to introduce the agents that have documented efficacy based on the current diagnostic gold standard, namely, liver biopsy. Although biopsy represents the most valid results, it is an expensive method that exposes patients to particular risks due to its invasive nature. Additionally, biopsy cannot represent the status of entire liver tissue [86].

These limitations have made the use of biopsy for drug screening trials and large-scale studies a less preferred option, blunting the pace of the reliable discovery of new drugs for NAFLD/NASH through clinical studies. Inevitably, a less invasive, low-cost, and noninvasive diagnostic method is necessary. When considering NAFLD and NASH as multifactorial diseases, no sole alternative indicator could reliably predict the clinical outcomes or therapeutic beneficial effects [87]. Recently, developments in multi-omics analyses have provided new insights into the pathogenesis of diseases such as NAFLD and NASH. Particularly, advanced integrated analysis of serum/liver cellular lipids in patients with NAFLD has revealed substantial metabolic pathways implicated in disease progression. These surrogate markers have considerable potential to identify risk factors and contribute to the monitoring of treatments for NAFLD/NASH [87,88].

## Conflict of interest

DPM has given talks and attended conferences sponsored by Amgen, Libytec, and Novo Nordisk. Dr. Mantzoros has been a shareholder of and reports grants through his institution and personal consulting fees from Coherus Inc and Pangea Inc; reports grants through his institution and personal consulting fees from Esai and Novo Nordisk; reports personal consulting fees and in kind support with research reagents from Ansh Inc; reports personal consulting fees from Genfit, P.E.S., Astra Zeneca, Aegerion, and Regeneron; reports in kind support (educational activity meals at and through his institution) from Amarin, Jansen, and Boehringer Ingelheim; and in kind support and consulting fees from the California Walnut Commission.
